# Dietary restriction reduces angiogenesis and growth in an orthotopic mouse brain tumour model

**DOI:** 10.1038/sj.bjc.6600298

**Published:** 2002-05-03

**Authors:** P Mukherjee, M M El-Abbadi, J L Kasperzyk, M K Ranes, T N Seyfried

**Affiliations:** Biology Department, Boston College, Chestnut Hill, Massachusetts, MA 02467, USA

**Keywords:** glioma, glycolysis, inflammation, energy metabolism, caloric restriction, microenvironment

## Abstract

Diet and lifestyle produce major effects on tumour incidence, prevalence, and natural history. Moderate dietary restriction has long been recognised as a natural therapy that improves health, promotes longevity, and reduces both the incidence and growth of many tumour types. Dietary restriction differs from fasting or starvation by reducing total food and caloric intake without causing nutritional deficiencies. No prior studies have evaluated the responsiveness of malignant brain cancer to dietary restriction. We found that a moderate dietary restriction of 30–40% significantly inhibited the intracerebral growth of the CT-2A syngeneic malignant mouse astrocytoma by almost 80%. The total dietary intake for the *ad* *libitum* control group (*n*=9) and the dietary restriction experimental group (*n*=10) was about 20 and 13 Kcal day^−1^, respectively. Overall health and vitality was better in the dietary restriction-fed mice than in the *ad libitum*-fed mice. Tumour microvessel density (Factor VIII immunostaining) was two-fold less in the dietary restriction mice than in the *ad libitum* mice, whereas the tumour apoptotic index (TUNEL assay) was three-fold greater in the dietary restriction mice than in the *ad libitum* mice. CT-2A tumour cell-induced vascularity was also less in the dietary restriction mice than in the *ad libitum* mice in the *in vivo* Matrigel plug assay. These findings indicate that dietary restriction inhibited CT-2A growth by reducing angiogenesis and by enhancing apoptosis. Dietary restriction may shift the tumour microenvironment from a proangiogenic to an antiangiogenic state through multiple effects on the tumour cells and the tumour-associated host cells. Our data suggest that moderate dietary restriction may be an effective antiangiogenic therapy for recurrent malignant brain cancers.

*British Journal of Cancer* (2002) **86**, 1615–1621. DOI: 10.1038/sj/bjc/6600298 www.bjcancer.com

© 2002 Cancer Research UK

## 

About 35 000 people in the United States are diagnosed each year with primary or secondary brain tumours (Black, 1991). The prognosis for many of these patients is poor despite new developments in neurosurgery, chemotherapy, and radiotherapy (Shapiro, 1999). Moreover, while the incidence of many cancers is decreasing, the incidence of brain cancer is increasing in both children and the elderly (Lowry *et al*, 1998; Kaiser, 1999; McKinley *et al*, 2000). The highly infiltrative growth of malignant brain tumours and difficulties in drug penetration of the neural parenchyma have limited therapeutic options. Hence, there is a crucial need for new and better brain tumour therapeutic strategies.

Several studies suggest that differences in diet and lifestyle can have major effects on tumour incidence, prevalence, and natural history (Blowers *et al*, 1997; Kaplan *et al*, 1997; Hu *et al*, 1999). Dietary restriction (DR) has long been recognised as a natural therapy that improves health, promotes longevity, and significantly reduces both the incidence and growth of many tumour types (Rous, 1914; Tannenbaum, 1959; Weindruch and Walford, 1988; Birt *et al*, 1999; Kritchevsky, 1999b). Dietary restriction differs from severe fasting or starvation in that it reduces total caloric or energy intake without causing deficiencies of any specific nutrients (Tannenbaum, 1959; Mukherjee *et al*, 1999a). The mechanisms by which DR reduces tumour growth are not yet clear, but likely involve changes in tumour cells and in tumour associated host cells.

Rous first suggested that DR might inhibit tumour growth by delaying host-mediated tumour vascularisation (Rous, 1914). Pili *et al* (1994) and Mukherjee *et al* (1999a) later provided direct support for Rous' hypothesis by showing that DR was antiangiogenic in experimental sarcomas and prostate tumours, respectively. Moreover, the antiangiogenic effect of DR was observed whether the calories were derived from fats or carbohydrates suggesting that tumour angiogenesis may be more sensitive to reductions in the amount rather than in the type of calories (Mukherjee *et al*, 1999a). Reduced total energy intake through DR may inhibit tumour growth by shifting tumour-host cell interactions from a proangiogenic to an antiangiogenic state.

Since neural tissues utilise glucose as the main energy substrate (Clarke and Sokoloff, 1999), brain tumours may be responsive to dietary and nutritional therapies. Moreover, the reliance of brain tumours on glycolysis for energy should make them especially vulnerable to DR, as DR shifts energy metabolism from glucose to ketone utilisation (Oudard *et al*, 1997; Greene *et al*, 2001). With the exception of an anecdotal report on the potential efficacy of a ketogenic diet toward paediatric astrocytoma (Nebeling *et al*, 1995), no studies have been performed to our knowledge on the effects of DR as a therapeutic intervention for brain tumours. In this study, we show for the first time that moderate DR can inhibit growth and vascularisation and enhance apoptosis in an orthotopic mouse brain tumour model. A preliminary account of these findings has appeared (Mukherjee *et al*, 2001).

## MATERIALS AND METHODS

### Mice

Mice of the C57BL/6J strain and the BALBc/J-SCID (severe combined immuno deficiency) strain were obtained from the Jackson laboratory (Bar Harbor, ME, USA). The mice were propagated in the animal care facility of the department of Biology of Boston College, using animal husbandry conditions described previously (Flavin *et al*, 1991). Male mice (8–10 weeks of age) were used for the studies and were provided with food either *ad libitum* (AL) or under restricted conditions (as below). Water was provided AL to all mice. The animal room was maintained at 22±1°C and cotton nesting pads were provided for additional warmth. All animal experiments were carried out with ethical committee approval in accordance with the National Institutes of Health Guide for the Care and Use of Laboratory Animals and were approved by the Institutional Care Committee. Also, these procedures meet the standards required by the UKCCCR guidelines (Workman *et al*, 1998).

### Brain tumour model

The syngeneic CT-2A experimental mouse brain tumour used for these studies was generated in our laboratory after implantation of 20-methylcholanthrene into the cerebral cortex of a C57BL/6J mouse according to the procedure of Zimmerman (Zimmerman and Arnold, 1941; Seyfried *et al*, 1992). Histologically, the CT-2A brain tumour is broadly classified as a poorly differentiated highly malignant anaplastic astrocytoma (Seyfried *et al*, 1992). The tumour grows orthotopically as a soft, noncohesive, and highly vascularised mass.

### Intracerebral tumour implantation

The CT-2A tumour was implanted into the cerebral cortex of C57BL/6J mice using a trocar as we previously described (Seyfried *et al*, 1987; Ranes *et al*, 2001). Briefly, mice were anaesthetised with pentobarbital (Vet Labs, Inc) intra-peritoneally and their heads were shaved and swabbed with 70% ethyl alcohol under sterile conditions. Small CT-2A tumour pieces (about 1 mm^3^) from a C57BL/6J donor mouse were implanted into the right cerebral hemisphere of anaesthetised recipient mice as we recently described (Ranes *et al*, 2001). All of the mice recovered from the surgical procedure and were returned to their cages when fully active. Initiation of tumours from intact tumour pieces is preferable to initiation from cultured cells since the pieces already contain an established microenvironment that facilitates tumour growth.

### Dietary restriction

The mice were group housed prior to the initiation of the experiment and were then separated and randomly assigned to either a control group that was fed AL or to an experimental group that was fed a total DR of 30% (70% of the control group). Each mouse was housed singly in a plastic shoe box cage with a filter top and was given a cotton nesting pad for warmth. Dietary restriction was initiated 7 days prior to tumour implantation and was continued for either 11 or 14 days after implantation. Total DR maintains a constant ratio of nutrients to energy, i.e., the average daily food intake (grams) for the AL fed mice was determined every other day and the DR-fed mice were given 70% of that quantity on a daily basis (Mukherjee *et al*, 1999a). All mice received PROLAB chow (Agaway Inc.), which contains a balance of mouse nutritional ingredients and, according to the manufacturer's specification, delivers 4.4 Kcal g^−1^ gross energy. Body weights of all mice were recorded every other day.

### Tumour growth

Intracerebral tumour growth was analysed directly by measuring total tumour dry weight. Tumours were dissected from normal appearing brain tissue, were frozen, and were then lyophilised to remove water. From our experience, total tumour dry weight is a more accurate measure of tumour growth than total wet weight because individual CT-2A tumours can vary in the degree of haemorrhage and oedema.

### Histology

Tumour samples were fixed in 10% neutral buffered formalin (Sigma) and embedded in paraffin. Tumours were sectioned at 5 um, stained with haematoxylin and eosin, and examined by light microscopy.

### Factor VIII staining and microvessel quantitation

After deparaffinisation, rehydration, and washing, the tumour sections were incubated with trypsin at 37°C for 30 min as we recently described for prostate tumours (Mukherjee *et al*, 1999a). Briefly, the sections were quenched with 0.3% H_2_O_2_-methanol for 30 min and then blocked with 10% normal goat serum in PBA buffer (100 ml of 0.01 M phosphate buffer with 0.9% sodium chloride, and 1.0 g bovine serum albumin and 0.1 ml Tween 20, pH 7.4). The sections were treated with rabbit polyclonal antibody directed against human factor VIII-related antigen (Dako Corp., Carpinteria, CA, USA; 1 : 100 dilution with PBA) followed by a biotinylated anti-rabbit IgG at 1 : 100 dilution (Vector Laboratories, Inc., Burlingame, CA, USA). The sections were then treated with avidin-biotin complex followed by 3-3′ diaminobenzidine as substrate for staining according to the manufacturer's directions (Vectastain Elite ABC kit ; Vector Laboratories, Inc.). The sections were then rinsed three times with PBS (0.01 M phosphate buffer with 0.9% NaCl). Sections were counter stained with methyl green and mounted. Corresponding tissue sections without primary antibody served as negative controls. Microvessel density was quantified by examining areas of vascular hotspots as previously described by Weidner *et al* (1991) with some modifications. Sections were scanned at low magnification (40× and 100×) for the localisation of vascular hotspots. The three most vascular areas of the tumour, not containing necrosis, were determined and then counted at higher magnification (200×). The values of the three sections were averaged and the results of three independent CT-2A tumours were analyzed. Branching structures were counted as a single vessel as previously shown (Mukherjee *et al*, 1999a).

### *In situ* apoptotic cell detection (TUNEL)

Apoptotic cells were detected using the ApopTag in situ detection kit TUNEL (terminal deoxynucleotidyl transferase mediated deoxyuridine triphosphate biotin nick end labelling) (Oncor, Gaithersberg, MD, USA) as we previously described (Mukherjee *et al*, 1999a). After deparaffinisation, rehydration and washing in PBS, the tissue sections were treated with proteinase K (20 μg ml^−1^) for 15 min at room temperature and were then washed in PBS. The sections were treated with 3% H_2_O_2_ in PBS for 5 min to quench endogenous peroxide activities. The 3′ hydroxy DNA strand breaks were enzymatically labelled with digoxygeninnucleotide via TdT and incubated for 1 h at 37°C. The reaction was terminated with stop buffer according to the manufacturer's protocol. Sections were then treated with anti-degoxygenin peroxidase for 30 min at room temperature, washed, stained with 3-3′ diaminobenzidine substrate, counter stained with hematoxylin, and finally were mounted. Tissue sections of post weaning normal female mouse mammary glands, provided by Oncor, were used as a positive control and staining of a corresponding tissue section without added TdT served as the negative control.

The apoptotic index was expressed as AI%=A×100/(A+C), where A=TUNEL positive cells and C=counter stained unlabelled cells. The tumour sections were scanned at lower magnification (40× and 100×) for nonnecrotic areas and approximately 2000 total cells were counted for each section at higher magnification (400×). The values of the three sections were averaged and the results of three independent CT-2A tumours were analysed.

### Proliferation index

Proliferation index measured the fraction of cells with proliferating cell nuclear antigen (PCNA) staining as we previously described (Mukherjee *et al*, 1999a). After deparaffinisation, rehydration and washing, the tissue sections were soaked in 10 mM citrate buffer (pH 6.0). The sections were heated in a microwave oven for 15 min (defrost cycle) and then cooled to room temperature to unmask the PCNA. Sections were then stained by the same procedures as described above except we used 10% horse serum as blocking agent and PCNA mouse monoclonal antibody (Dako) as the primary antibody. Light microscopy (400×) was used to count both PCNA positive proliferating cells and total tumour cells in three non necrotic areas of each tissue section as previously shown (Mukherjee *et al*, 1999a).

### *In vivo* Matrigel model of angiogenesis

Male BALB/c-SCID mice were divided into two groups of three mice each: a control AL group and a 30% DR group. Dietary restriction treatment was initiated 7 days prior to tumour cell injection. CT-2A tumour cells were grown in culture and harvested with 0.25% trypsin containing 1 mM EDTA. The cells were washed twice, resuspended in serum free DMEM, and then thoroughly mixed with Matrigel (Collaborative Biomedical) 1 : 2 (v v^−1^) at 4°C as we recently described (Manfredi *et al*, 1999). Mice were anaesthetised with Isovet (Schering Plough Animal Health, Omaha, NE, USA) and then injected with 1×10^6^ cells in 300 μl of Matrigel subcutaneously in the dorsal midline using a prechilled tuberculin syringe (27 gauge needle). The mice were maintained for another 7 days under the dietary regime at which time they were euthanised and the Matrigel plug with the surrounding skin was removed as we previously described (Manfredi *et al*, 1999). Vascularity was photographed using a dissecting photomicroscope (Leica, WILD macroskop).

## RESULTS

No adverse effects were seen in the mice maintained on the 30–40% DR. Despite a reduction in total body weight, the DR-fed mice appeared healthy and were more active than the AL-fed mice as assessed by ambulatory and grooming behaviour. No signs of vitamin or mineral deficiency were observed in the DR-fed mice according to standard criteria for mice (Hoag and Dickie, 1968). These findings are consistent with the well-recognized health benefits of mild to moderate diet restriction in rodents (Weindruch and Walford, 1988; Keenan *et al*, 1999).

### Energy Intake

**Figure 1:**
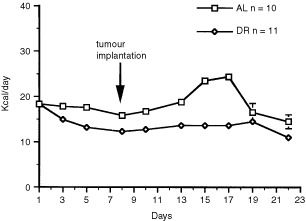
Energy intake in male C57BL6/J mice bearing the intracerebral CT-2A brain tumour. DR was initiated on day 1 and tumours were implanted on day 8. Values are expressed as means±s.e.m. and *n*=the number of tumour-bearing mice examined in each group.

Our experimental design involved pretreatment with DR for 7 days prior to intracerebral tumour implantation. This was done to separate the physiological stress of single cage housing and food restriction from surgical brain trauma. A slight reduction in energy intake was noticed in both the AL-fed and the DR-fed mice at the initiation of the experiment (Figure 1

). This was attributed to the effects of moving the mice from group housing to single cage housing.

Energy intake increased significantly in the AL-fed group about 2 days after intracerebral tumour implantation. This resulted from a period of hyperphagia likely associated with cerebral hyperglycolysis following the traumatic injury of tumour implantation (Bergsneider *et al*, 1997). Energy intake was not increased in the DR-fed mice to compensate for hyperphagia. Consequently, energy intake was indirectly reduced in the DR-fed mice from 30% to about 40% of that in the control AL-fed group. Hyperphagia associated with orthotopic brain tumour growth is a novel finding and was not observed in C57BL/6J mice with the CT-2A tumour grown subcutaneously in flank (Ranes *et al*, 2001).

The total energy intake of the AL-fed group was about 18 Kcal day^−1^, but rose to about 24 Kcal day^−1^ during the hyperphagic period (Figure 1). The total energy intake of the DR-fed group was adjusted to 13 Kcal day^−1^ during the 22 day experiment. The DR-fed mice lost about 12% of their body weight during the first week of treatment and their weights remained significantly lower than those of the AL group throughout the study. The mean (±s.e.m.) body weights (g) of the AL and DR mice after 17 days of treatment were 23.6±0.6 and 20.3±0.4, respectively (*P*<0.01, two tailed *t*-test). Total energy intake and body weights dropped after 17 days in the AL-fed group due to increased tumour burden.

### DR reduced intracerebral CT-2A tumour growth

**Figure 2:**
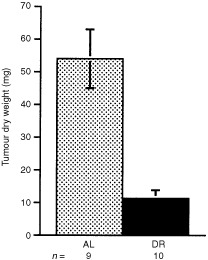
Influence of DR on the intracerebral growth of the CT-2A brain tumour. DR was initiated 7 days before tumour implantation and was continued for 14 days after implantation as shown in Figure 1. Values are expressed as means±s.e.m. and *n*=the number of tumour-bearing mice examined in each group. The dry weight of the treated tumours was significantly lower than that of the control tumours (*P*<0.001, two tailed *t*-test).

Dry weights of the intracerebral CT-2A tumours were approximately 79.5% lower in the DR-fed mice than in the AL-fed mice (Figure 2

). It is important to mention that all implanted tumours grew in both the AL and DR groups. These findings indicate that DR did not prevent tumour take, but significantly reduced intracerebral growth of the malignant CT-2A brain tumour. We do not think the reduced food intake beyond day 19 in the control AL mice reduced the difference in tumor size between the AL and CR mice.

### Influence of DR on tumour vascularity, apoptosis, and cell proliferation

**Figure 3:**
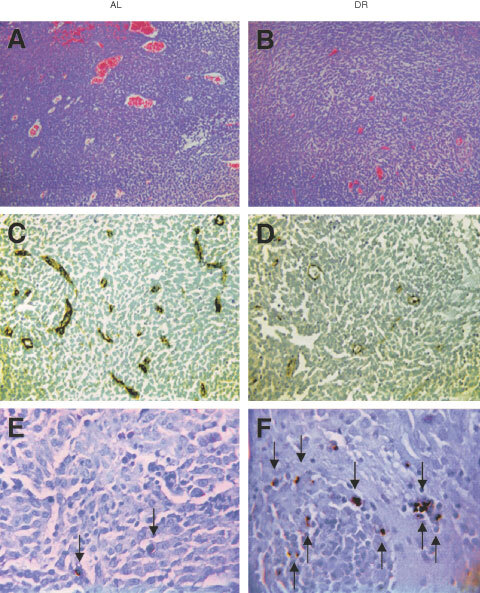
Influence of DR on microvessel density and apoptosis in the CT-2A brain tumour. DR was initiated 7 days before intracerebral tumour implantation and was continued for 11 days. H&E stained tumour sections in an AL mouse (**A**) and in a DR mouse (**B**) (100×). Factor VIII immunostaining from the tumour grown in an AL mouse (**C**) and in a DR mouse (**D**) (200×). TUNEL positive apoptotic cells (arrows) from the tumour grown in an AL mouse (**E**) and in a DR mouse (**F**) (400×). Each stained section was representative of the entire tumour. All images were produced from digital photography.

We next examined tumour morphology and blood vessel densities using H&E staining and Factor VIII immunostaining to determine if DR influenced tumour angiogenesis. Three independent tumours from the AL and DR groups were chosen at random for these studies. The number and size of blood vessels and tumour cell density were noticeably less in the DR-fed mice than in the AL-fed mice (Figure 3A,B

). Also, the tumour microvessel density of the DR-fed mice was about half of that in the AL-fed mice (Figure 3C,D, and Table 1

**Table 1:**
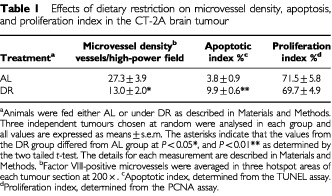
Effects of dietary restriction on microvessel density, apoptosis, and proliferation index in the CT-2A brain tumour

).

To determine if DR influenced programmed cell death (apoptosis) in the CT-2A tumour, we compared the number of TUNEL positive cells (apoptotic index) in the AL-fed and DR-fed mice. The apoptotic index was almost three-fold greater in the DR mice than in the AL mice (Figure 3E,F and Table 1). No significant difference was found, however, between the DR and AL mice for the PCNA proliferation index (Table 1), suggesting that the DR-induced reduction of CT-2A growth was not associated with reduced tumour cell proliferation.

### DR reduced vascularity in the *in vivo* Matrigel model of angiogenesis

**Figure 4:**
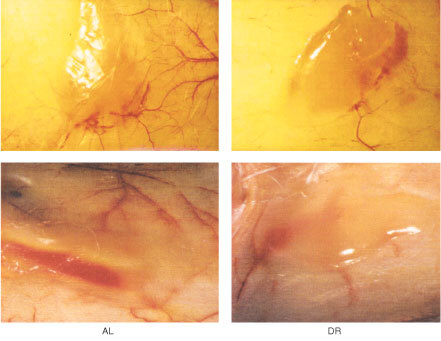
Tumour cell-induced vascularity in the *in vivo* Matrigel plug assay. CT-2A tumour cells in Matrigel were injected s.c. in the flank of a BALB/c-SCID mouse as described in Materials and Methods. DR treatment was initiated 7 days prior to tumour cell injection. The Matrigel plugs with surrounding skin were removed on day 7 after implantation and photographed at 12.5×.

The *in vivo* Matrigel angiogenesis model represents early events of angiogenesis and tumour progression and is dependent on activation and infiltration of host stromal cells which include monocytes, macrophages, and endothelial cell precursors (Manfredi *et al*, 1999). DR reduced vascularity when the CT-2A tumour cells were grown in the *in vivo* Matrigel model of angiogenesis (Figure 4

). Although blood vessel quantitation is difficult in the plugs, it is clear from the figure that both the number and dilation of vessels was noticeably less in and around the plugs from the DR-fed mice than from the AL-fed mice. Similar qualitative differences were seen in the other independent sample. These findings indicate that DR reduces the angiogenic properties of the CT-2A tumour cells whether grown within or outside of the central nervous system.

## DISCUSSION

We found that a moderate DR of 30–40% significantly reduced angiogenesis and growth of the CT-2A experimental mouse astrocytoma. Moreover, DR enhanced CT-2A cell apoptosis without effecting cell proliferation. Previous studies showed that moderate DR could reduce the growth of histologically diverse non-neural tumours (Rous, 1914; Tannenbaum, 1959; Kritchevsky, 1999a; Mukherjee *et al*, 1999a). Our studies are the first to document this phenomenon in a brain tumour model and suggest that brain tumours may be especially vulnerable to the growth-inhibitory effects of DR. It will be important to document the extent to which DR reduces angiogenesis and growth in other brain tumour models.

Despite a 12% reduction in body weight, the DR-fed mice were more active and healthy than the AL fed mice. Keenan and co-workers recently suggested that the AL feeding of sedentary rodents is a form of over feeding that can produce adverse health effects (Keenan *et al*, 1999). Our results support this contention since CT-2A tumour angiogenesis and growth was significantly greater in mice under AL feeding than under DR.

We found that angiogenic biomarkers may be useful for evaluating the influence of energy intake and nutrition on the growth and progression of experimental brain cancer. Moderate DR significantly reduced microvessel density, increased the apoptotic index, but had little effect on the PCNA labelling index in the CT-2A brain tumour. Other investigators have also reported that antiangiogenic growth factors and cytokines can reduce tumour microvessel density, increase apoptosis, but have little effect on cell proliferation (Holmgren *et al*, 1995; O'Reilly *et al*, 1996; Tanaka *et al*, 1997; Beecken *et al*, 2001). Our results therefore support previous findings that DR produces a pattern of biomarker changes similar to the changes seen following the implementation of antiangiogenic therapies (Mukherjee *et al*, 1999a; 1999b).

The mechanisms by which DR reduced CT-2A tumour angiogenesis and growth are not yet clear, but may involve effects on both the tumour cells and the tumour-associated host cells. It is documented that human and experimental gliomas are dependent on glycolysis for energy (Mies *et al*, 1990; Ikezaki *et al*, 1992; Oudard *et al*, 1997), and that DR-induced caloric restriction reduces glycolytic energy and down-regulates glycolytic gene expression (Lee *et al*, 2000; Cao *et al*, 2001; Greene *et al*, 2001). Additionally, the DR-induced down regulation of glycolysis should also reduce the level of pyruvic acid, a glycolytic end product with angiogenic activity (Lee *et al*, 2001).

Glucose is used exclusively for adult brain energy metabolism under normal physiological conditions, but the brain will metabolise ketone bodies for energy when blood glucose levels decrease as during fasting or DR (Clarke and Sokoloff, 1999; Greene *et al*, 2001). Since ketone bodies are metabolised directly to acetyl-CoA in the mitochondria, they bypass cytoplasmic glycolysis and provide energy directly through the Krebs cycle (Nehlig and Pereira de Vasconcelos, 1993; Clarke and Sokoloff, 1999). We recently showed that DR produces ketosis in epileptic mice and that the degree of ketosis is inversely proportional to blood glucose levels (Greene *et al*, 2001). Further studies will be needed to determine if reduced glycolytic energy and elevated ketosis underlie the antiangiogenic and growth inhibitory effects of DR.

In addition to possible effects on energy metabolism, DR may also reduce CT-2A angiogenesis and growth through effects on tumour associated host cells. The progression of human and experimental brain tumours is dependent to a large extent on the proangiogenic and inflammatory properties of activated glia and macrophages (Seyfried, 2001). Indeed, the degree of tumour angiogenesis and malignancy is generally correlated with the number and activation state of tumour-associated macrophages and microglia (Wood and Morantz, 1979; Roggendorf *et al*, 1996; Nishie *et al*, 1999; Polverini, 1999; Badie and Schartner, 2000). Recent studies also indicate that moderate DR reduces brain inflammation associated with ageing and neurodegeneration (Duan *et al*, 2001; Lee *et al*, 2000). Furthermore, dietary energy restriction can elevate glucocorticoid hormone that could further reduce tumour inflammation and growth through down regulation of stress-activated protein kinase pathways (Birt *et al*, 1999). Hence, DR may reduce CT-2A progression through a global down-regulation of inflammatory and angiogenic properties of the tumour microenvironment.

We also found that DR caused a noticeable reduction in the number and the dilation of blood vessels in the *in vivo* Matrigel model of angiogenesis indicating that DR can reduce angiogenesis both within and outside of the central nervous system. It is possible that DR reduces the inflammatory properties of tumour-associated host cells and thereby shifts tumour-host cell interactions from a proangiogenic to an antiangiogenic state. Studies are planned to test these possibilities.

Our findings may have relevance to those *in vivo* studies where food intake and body weight are reduced in conjunction with anticancer therapies or with cancer cachexia. Reduction of energy intake as a covariable of anorexic anticancer therapies may confound interpretation of results (Ranes *et al*, 2001). It would be important therefore to control for the antitumour effects of dietary reduction in the preclinical evaluation of new cancer drugs. Weight loss associated with cancer cachexia differs from weight loss associated with anorexia (reduction in food intake) since cachexia can occur without anorexia and is produced from factors released by the tumour (Tisdale, 2001). Although appearing counterintuitive, we suggest that DR may antagonise cachexia by reducing tumour size and therby reducing levels of procachexic factors.

Although DR is recognised as a preventative measure for carcinogenisis, it is clear from our findings on the the CT-2A brain tumour that DR is not a preventative intervention since all of the tumours implanted grew despite the 7 day DR pretreatment period. The DR-induced inhibition of CT-2A angiogenesis and growth suggests that DR retards tumour progression. Whether DR would also increase the survival time of CT-2A-tumour bearing mice is not clear. Survival studies are difficult with this rapidly growing brain tumour model since the tumour will grow through the implantation burr hole and then subcutaneously over the skull as we previously described for the EPEN model (Seyfried *et al*, 1987). This relieves intracranial pressure and artificially extends longevity. In humans with malignant brain tumours, it is the intracranial pressure that usually leads to morbidity.

In summary, we have demonstrated that DR alone can reduce angiogenesis and growth in an experimental mouse brain tumour. Moreover, the antitumour action of DR likely operates through multiple effects on the tumour cells and on the tumour associated host cells. We contend that our experimental protocol may have therapeutic potential for recurrent human gliomas since the time of surgical tumour resection in humans would be comparable to the time of tumour transplantation in mice. In other words, implementation of DR in the clinic could be most effective immediately following tumour removal and may delay tumour recurrence. Because DR is easy to administer and is devoid of adverse side effects, our preclinical studies suggest that DR or caloric restriction may have efficacy as a non-invasive therapy for recurrent malignant brain cancers.

## References

[bib1] BadieBSchartnerJM2000 Flow cytometric characterization of tumor-associated macrophages in experimental gliomas Neurosurgery 46 957961 10764271 10.1097/00006123-200004000-00035

[bib2] BeeckenWDFernandezAJoussenAMAchillesEGFlynnELoKMGilliesSDJavaherianKFolkmanJShingY2001 Effect of antiangiogenic therapy on slowly growing, poorly vascularized tumors in mice J Natl Cancer Inst 93 382387 11238700 10.1093/jnci/93.5.382

[bib3] BergsneiderMHovdaDAShalmonEKellyDFVespaPMMartinNAPhelpsMEMcArthurDLCaronMJKrausJFBeckerDP1997 Cerebral hyperglycolysis following severe traumatic brain injury in humans: a positron emission tomography study J Neurosurg 86 241251 9010426 10.3171/jns.1997.86.2.0241

[bib4] BirtDFYaktineADuysenE1999 Glucocorticoid mediation of dietary energy restriction inhibition of mouse skin carcinogenesis J Nutr 129 571S574S 10064335 10.1093/jn/129.2.571S

[bib5] BlackPM1991 Brain tumors. Part 1 N Engl J Med 324 14711476 1822669 10.1056/NEJM199105233242105

[bib6] BlowersLPreston-MartinSMackWJ1997 Dietary and other lifestyle factors of women with brain gliomas in Los Angeles County (California, USA) Cancer Causes Control 8 512 9051317 10.1023/a:1018437031987

[bib7] CaoSXDhahbiJMMotePLSpindlerSR2001 Genomic profiling of short- and long-term caloric restriction effects in the liver of aging mice Proc Natl Acad Sci USA 98 1063010635 11535822 10.1073/pnas.191313598 PMC58517

[bib8] ClarkeDDSokoloffL1999 Circulation and energy metabolism in the brainInBasic NeurochemistrySiegel GJ, Agranoff BW, Albers RW, Fisher SK, Uhler MD (eds)pp637669New York: Lippincott-Raven

[bib9] DuanWLeeJGuoZMattsonMP2001 Dietary restriction stimulates BDNF production in the brain and thereby protects neurons against excitotoxic injury J Mol Neurosci 16 112 11345515 10.1385/JMN:16:1:1

[bib10] FlavinHJWieraszkoASeyfriedTN1991 Enhanced aspartate release from hippocampal slices of epileptic (El) mice J Neurochem 56 10071011 1671582 10.1111/j.1471-4159.1991.tb02021.x

[bib11] GreeneAETodorovaMTMcGowanRSeyfriedTN2001 Caloric restriction inhibits seizure susceptibility in epileptic EL mice by reducing blood glucose Epilepsia 42 13711378 11879337 10.1046/j.1528-1157.2001.17601.x

[bib12] HoagWGDickieMM1968 NutritionInBiology of the Laboratory MouseGreen EL (ed)New York: Dover

[bib13] HolmgrenLO'ReillyMSFolkmanJ1995 Dormancy of micrometastases: balanced proliferation and apoptosis in the presence of angiogenesis suppression[see comments]Nat Med 1 149153 7585012 10.1038/nm0295-149

[bib14] HuJLa VecchiaCNegriEChatenoudLBosettiCJiaXLiuRHuangGBiDWangC1999 Diet and brain cancer in adults: a case-control study in northeast China Int J Cancer 81 2023 10077146 10.1002/(sici)1097-0215(19990331)81:1<20::aid-ijc4>3.0.co;2-2

[bib15] IkezakiKBlackKLConklinSGBeckerDP1992 Histochemical evaluation of energy metabolism in rat glioma Neurol Res 14 289293 1360622 10.1080/01616412.1992.11740072

[bib16] KaiserJ1999 No meeting of minds on childhood cancer Science 286 18321834 10610570 10.1126/science.286.5446.1832

[bib17] KaplanSNovikovIModanB1997 Nutritional factors in the etiology of brain tumors: potential role of nitrosamines, fat, and cholesterol Am J Epidemiol 146 832831 9384204 10.1093/oxfordjournals.aje.a009201

[bib18] KeenanKPBallamGCSoperKALaroquePColemanJBDixitR1999 Diet, caloric restriction, and the rodent bioassay Toxicol Sci 52 2434 10630587 10.1093/toxsci/52.2.24

[bib19] KritchevskyD1999a Caloric restriction and experimental carcinogenesis Toxicol Sci 52 1316 10630585 10.1093/toxsci/52.2.13

[bib20] KritchevskyD1999b Fundamentals of nutrition: applications to cancer researchInNutritional OncologyHeber D, Blackburn GL, Go VLW (eds)pp510Boston: Academic Press

[bib21] LeeCKWeindruchRProllaTA2000 Gene-expression profile of the ageing brain in mice Nat Genet, 25 294297 10888876 10.1038/77046

[bib22] LeeMSMoonEJLeeSWKimMSKimKWKimYJ2001 Angiogenic activity of pyruvic acid in in vivo and in vitro angiogenesis models Cancer Res 61 32903293 11309282

[bib23] LowryJKSnyderJJLowryPW1998 Brain tumors in the elderly: recent trends in a Minnesota cohort study Arch Neurol 55 922928 9678309 10.1001/archneur.55.7.922

[bib24] ManfrediMGLimSClaffeyKPSeyfriedTN1999 Gangliosides influence angiogenesis in an experimental mouse brain tumor Cancer Res 59 53925397 10537325

[bib25] McKinleyBPMichalekAMFenstermakerRAPlunkettRJ2000 The impact of age and sex on the incidence of glial tumors in New York state from 1976 to 1995 J Neurosurg 93 932939 11117865 10.3171/jns.2000.93.6.0932

[bib26] MiesGPaschenWEbhardtGHossmannKA1990 Relationship between of blood flow, glucose metabolism, protein synthesis, glucose and ATP content in experimentally-induced glioma (RG1 2.2) of rat brain J Neurooncol 9 1728 2213113 10.1007/BF00167064

[bib27] MukherjeePEl-AbbadiMMKasperzykJLSeyfriedTN2001 Caloric restriction reduces growth and angiogenesis in a mouse brain tumor Proc Amer Assoc Cancer Res 42 651652

[bib28] MukherjeePSotnikovAVMangianHJZhouJRVisekWJClintonSK1999a Energy intake and prostate tumor growth, angiogenesis, and vascular endothelial growth factor expression J Natl Cancer Inst 91 512523 10088621 10.1093/jnci/91.6.512

[bib29] MukherjeePZhauJ-RSotnikovAVClintonSK1999b Dietary and nutritional modulation of tumor angiogenesisInAntiangiogenic Agents in Cancer TherapyTeicher BA (ed)pp237261Totowa, NJ: Humana Press

[bib30] NebelingLCMiraldiFShurinSBLernerE1995 Effects of a ketogenic diet on tumor metabolism and nutritional status in pediatric oncology patients: two case reports J Am Coll Nutr 14 202208 7790697 10.1080/07315724.1995.10718495

[bib31] NehligAPereira de VasconcelosA1993 Glucose and ketone body utilization by the brain of neonatal rats Prog Neurobiol 40 163221 8430212 10.1016/0301-0082(93)90022-k

[bib32] NishieAOnoMShonoTFukushiJOtsuboMOnoueHItoYInamuraTIkezakiKFukuiMIwakiTKuwanoM1999 Macrophage infiltration and heme oxygenase-1 expression correlate with angiogenesis in human gliomas Clin Cancer Res 5 11071113 10353745

[bib33] O'ReillyMSHolmgrenLChenCFolkmanJ1996 Angiostatin induces and sustains dormancy of human primary tumors in mice Nat Med 2 689692 8640562 10.1038/nm0696-689

[bib34] OudardSBoitierEMiccoliLRoussetSDutrillauxBPouponMF1997 Gliomas are driven by glycolysis: putative roles of hexokinase, oxidative phosphorylation and mitochondrial ultrastructure Anticancer Res 17 19031911 9216643

[bib35] PiliRGuoYChangJNakanishiHMartinGRPassanitiA1994 Altered angiogenesis underlying age-dependent changes in tumor growth J Natl Cancer Inst 86 13031314 7520508 10.1093/jnci/86.17.1303

[bib36] PolveriniPJ1999 Contribution of the extracellular matrix and macrophages in angiogenesisInAntiangiogenic Agents in Cancer TherapyTeicher BA (ed)pp6575Totowa, New Jersey: Humana Press

[bib37] RanesMKEl-AbbadiMManfrediMGMukherjeePPlattFMSeyfriedTN2001 N -butyldeoxynojirimycin reduces growth and ganglioside content of experimental mouse brain tumours Br J Cancer 84 11071114 11308262 10.1054/bjoc.2000.1713 PMC2363859

[bib38] RoggendorfWStruppSPaulusW1996 Distribution and characterization of microglia/macrophages in human brain tumors Acta Neuropathol 92 288293 8870831 10.1007/s004010050520

[bib39] RousP1914 The influence of diet on transplanted and spontaneous mouse tumors J Exp Med 20 433451 19867833 10.1084/jem.20.5.433 PMC2125200

[bib40] SeyfriedTN2001 Perspectives on brain tumor formation involving macrophages, glia, and neural stem cells Perspect Biol Med 44 263282 11370160 10.1353/pbm.2001.0035

[bib41] SeyfriedTNEl-AbbadiMRoyML1992 Ganglioside distribution in murine neural tumors Mol Chem Neuropathol 17 147167 1418222 10.1007/BF03159989

[bib42] SeyfriedTNYuRKSaitoMAlbertM1987 Ganglioside composition of an experimental mouse brain tumor Cancer Res 47 35383542 3581087

[bib43] ShapiroWR1999 Current therapy for brain tumors: back to the future Arch Neurol 56 429432 10199330 10.1001/archneur.56.4.429

[bib44] TanakaTManomeYWenPKufeDWFineHA1997 Viral vector-mediated transduction of a modified platelet factor 4 cDNA inhibits angiogenesis and tumor growth Nat Med 3 437442 9095178 10.1038/nm0497-437

[bib45] TannenbaumA1959 Nutrition and cancerInPhysiopathology of CancerHomburge F (ed)pp517562NY: Paul B Hober

[bib46] TisdaleMJ2001 Cancer anorexia and cachexia Nutrition 17 438442 11377146 10.1016/s0899-9007(01)00506-8

[bib47] WeidnerNSempleJPWelchWRFolkmanJ1991 Tumor angiogenesis and metastasis – correlation in invasive breast carcinoma N Engl J Med 324 18 10.1056/NEJM199101033240101 1701519

[bib48] WeindruchRWalfordRL1988 The retardation of aging and disease by dietary restriction.Springfield, IL: Thomas

[bib49] WoodGWMorantzRA1979 Immunohistologic evaluation of the lymphoreticular infiltrate of human central nervous system tumors J Natl Cancer Inst 62 485491 216840 10.1093/jnci/62.3.485

[bib50] WorkmanPTwentymanPBalkwillFBalmainAChaplinDDoubleJEmbletonJNewellDRaymondRStablesJStephensTWallaceJ1998 United Kingdom Co-ordinating Committee on Cancer Research (UKCCCR) Guidelines for the Welfare of Animals in Experimental Neoplasia (Second Edition) Br J Cancer 77 110 10.1038/bjc.1998.1 PMC2151254 9459138

[bib51] ZimmermanHMArnoldH1941 Experimental brain tumors: I. tumors produced with methylcholanthrene Cancer Res 1 919938

